# Role of nucleus accumbens dopamine 2 receptors in motivating cocaine use in male and female rats prior to and following the development of an addiction-like phenotype

**DOI:** 10.3389/fphar.2023.1237990

**Published:** 2023-07-26

**Authors:** Eleanor Blair Towers, Ivy L. Williams, Emaan I. Qillawala, Wendy J. Lynch

**Affiliations:** ^1^ Psychiatry and Neurobehavioral Sciences, University of Virginia, Charlottesville, VA, United States; ^2^ Medical Scientist Training Program, University of Virginia, Charlottesville, VA, United States

**Keywords:** sex differences, dopamine 2 receptor, addiction-like phenotype, cocaine intake, extended-access, self-administration, enhanced motivation

## Abstract

A hallmark of cocaine use disorder (CUD) is dysfunction of dopamine signaling in the mesolimbic pathway, including impaired dopamine 2 (D2) receptor signaling. One of the most replicated findings in human imagining studies is decreased striatal D2 receptor binding in individuals with a substance use disorder relative to healthy controls; however, the vast majority of the data is from males, and findings in smokers suggest this molecular shift may not translate to females. The goal of this study was to determine whether there are sex differences in the role of D2 receptors in motivating cocaine use prior to and following the development of an addiction-like phenotype (defined by an enhanced motivation for cocaine relative to the short-access, ShA, group). Here, male and female rats were given ShA (20 infusions/day, 3 days) or extended-access (ExA; 24h/day, 96 infusions/day, 10 days) to cocaine self-administration and then following 14 days of withdrawal, were tested under a progressive-ratio schedule to assess motivation for cocaine use. Once a stable level of motivation was established, the effect of NAc-infusions of the D2 receptor antagonist eticlopride (0–3.0 µg/side) were examined. We found that in males, eticlopride was less effective at decreasing motivation for cocaine following ExA *versus* ShA self-administration, particularly at low eticlopride doses. In contrast, in females, there were no differences in the effectiveness of eticlopride between ExA and ShA. These findings indicate that males, but not females, become less sensitive to NAc-D2 receptor antagonism with the development of an addiction-like phenotype.

## 1 Introduction

A hallmark of cocaine use disorder (CUD) is dysfunction of dopamine signaling in the mesolimbic pathway, including impaired dopamine 2 (D2) receptor signaling. This was first reported in humans by Volkow and others (1990) using positron emission tomography (PET) and showed that individuals with CUD had lower striatal D2 receptor availability compared to healthy controls. Since the original report with cocaine (Volkow et al., 1990), this neuroadaptation has been observed in many subsequent studies for cocaine and other drugs such as methamphetamine, nicotine, opioids, and alcohol (Volkow et al., 1993, 2001; Martinez et al., 2004, 2005, 2012; Fehr et al., 2008). Low levels of striatal D2 receptor availability have also been shown to be associated with greater drug craving and relapse vulnerability in individuals with substances use disorders (Heinz et al., 2004; Wang et al., 2011). Moreover, high levels of striatal D2 receptor availability has been observed in unaffected members of families with a history of alcohol use disorder, suggesting that higher striatal D2 receptor availability is protective against the development of substance use disorder (Volkow et al., 2006).

In parallel, preclinical studies have shown that subordinate male cynomolgus monkeys have lower D2 receptor availability in the basal ganglia, which includes the ventral striatum (nucleus accumbens; NAc), and are more sensitive to the reinforcing effects of cocaine as compared to dominant monkeys (Morgan et al., 2002). Studies in male rats further indicate that changes in D2 receptors occur as the result of chronic self-administration. For example, Conrad and colleagues (2010) showed that surface expression of D2 receptors in the NAc decreased following prolonged cocaine self-administration (4h/day for 3 weeks). Similarly, Mateo et al., 2005 showed that NAc dopamine terminals and presynaptic D2 receptors (autoreceptors) are less sensitive to cocaine following extended-access (ExA; 24-h/d for 10 days) cocaine self-administration *versus* acute cocaine administration (1.5 mg/kg, i. v.). Our recent findings in male rats further indicate that there is a functional shift in D2 receptor signaling in the reward pathway following the development of an addiction-like phenotype. We have used an enhanced motivation for cocaine, as assessed under a progressive-ratio (PR) schedule, to define the development of an addiction-like phenotype since this feature develops following withdrawal from ExA (24-h/day for 10 days), but not ShA (20 infusions/day for 5 days) cocaine self-administration, and like in humans, once this feature emerges, it represents a relatively permanent shift to a higher motivational state (Ramôa et al., 2013, 2014; Doyle et al., 2014; Bakhti-Suroosh et al., 2019). The development of an enhanced motivation for cocaine also corresponds to the development of other addiction-like features, including compulsive cocaine use despite negative consequences (Towers et al., 2021) and enhanced cocaine-seeking (e.g., Peterson et al., 2014; Towers et al., 2023). Furthermore, we showed that in males the development of an enhanced motivation for cocaine is accompanied by a decreased sensitivity to NAc D2 receptor antagonism (relative to ShA controls), indicating that the role of D2 receptor signaling in motivating cocaine use becomes diminished with the development of addiction (Lynch et al., 2021).

One major caveat is that the evidence for changes in D2 receptor signaling with the development of addiction has been derived almost entirely from men and male animals. This is important particularly considering an accumulating body of evidence indicating that the mechanisms that underlie addiction are different in males and females. For example, while both male and female subordinate cynomolgus monkeys are less vulnerable to the reinforcing effects of cocaine as compared to their dominant counterparts, in females, this protection corresponds to lower D2 receptor availability (Nader et al., 2012), suggesting that the relationship between D2 receptor availability and vulnerability to cocaine is opposite in males *versus* females. Even more notable for this study are data from human smokers showing that males have reduced striatal D2 receptor availability compared to healthy controls, but within females, striatal D2 receptor availability is comparable between smokers and health controls (Brown et al., 2012). Similar sex differences have also been reported for cortical D2 receptor availability (Zakiniaeiz et al., 2019). These findings are intriguing, particularly considering that this neuroadaptation is thought to reflect poorer treatment outcomes and greater addiction severity (Volkow et al., 1999), yet it is not observed in females who show worse treatment outcome and greater addiction severity than males (for review see Towers et al., 2023). Therefore, sex differences in the molecular shifts that occur with D2 receptor signaling with the development of addiction needs to be further investigated.

Thus, the purpose of this study was to determine whether there are sex differences in the role of NAc D2 receptors in motivating cocaine use prior to and following the development of an addiction-like phenotype (defined by an enhanced motivation for cocaine relative to the ShA group). As in our previous study in males, shifts in D2 receptor signaling were determined by comparing effects of intra-NAc D2 receptor antagonism on PR responding for cocaine following withdrawal from either ShA cocaine self-administration (which does not induce an enhanced motivation for cocaine from baseline; Roberts et al., 2007) or ExA cocaine self-administration (which induces an enhanced motivation for cocaine from baseline; Towers et al., 2021). Based on the clinical finding in smokers, we predicted that the role of NAc-D2 receptors would become diminished with the development of an addiction-like phenotype in males, but not females.

## 2 Methods

### 2.1 Subjects

Sexually mature male (*N* = 39) and female (*N* = 36) Sprague-Dawley rats (Charles River Laboratories, ME) were used as subjects. Behavioral data from a subset of the male rats (N = 28) has previously published (Lynch et al., 2021) and was included here for comparison to the female behavioral data. The male and female subjects included in the present study were run contemporaneously. Rats were individually housed in operant test chambers (Med Associates Inc., VT) in a humidity (40%–70%) and temperature (20–22°C) controlled vivarium that was maintained on a 12-h light/dark cycle (room/house lights on at 7 AM) with *ad libitum* access to water and food (Teklad LM-485 7,912; except as noted below for some rats during cocaine self-administration training). To ensure rapid rates of acquisition of cocaine self-administration, rats were pre-trained to lever-press for sucrose pellets (45 mg; fixed-ratio 1 (FR1) schedule using methods previously described (Doyle et al., 2014; Ramôa et al., 2014). The health of the rats was monitored daily over the course of the study, which included daily observation and weighing the rats at least three times a week. All the procedures were conducted within the guidelines set by the National Institutes of Health and approved by the University of Virginia Animal Care and Use Committee.

### 2.2 Procedures

#### 2.2.1 Surgeries and catheter maintenance

Rats underwent a jugular catheterization surgery using methods previously described (Doyle et al., 2014; Ramôa et al., 2014). The catheters were maintained by flushing with heparinized saline 3 days a week, which also helped verify patency throughout the study, and methohexital (1.5 mg/kg) was used to confirm patency when necessary. If a right catheter failed, a new catheter was implanted into the left jugular vein with testing resuming after 2-day of recovery. Rats were also implanted with a bilateral infusion cannula aimed at the NAc core (+1.2 mm anterior-posterior, ± 1.5 mm mediolateral, -5.7 mm dorsoventral) during the time of jugular catheterization or the second half of the abstinence period (Doyle et al., 2014; Ramôa et al., 2014; Lynch et al., 2021). This region was targeted because it is known to integrate dopamine and glutamate signaling and to mediate the reinforcing and motivational properties of addictive drugs (Kalivas and McFarland, 2003; Doyle et al., 2014; Ramôa et al., 2014; Lynch et al., 2021). Rats were given ketoprofen (2 mg/kg, subcutaneously) for pain management 1 day post-operatively and gentamicin (4 mg/kg, intravenously) to reduce risk of infection 2 days post-operatively for all jugular and cannula surgeries.

#### 2.2.2 Cocaine self-administration

**FIGURE 1:**
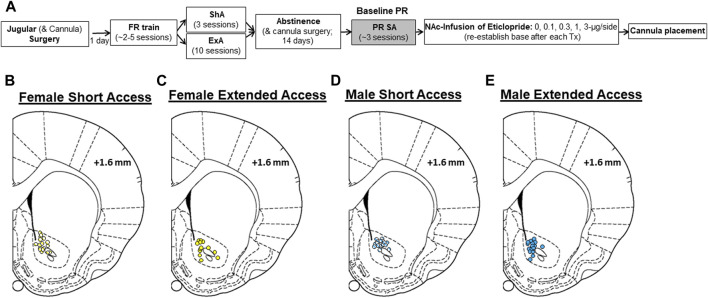
Summary of experimental design and cannulae placement. **(A)** Male and female rats were implanted with jugular catheters and bilateral infusion cannula aimed at the nucleus accumbens (NAc; some rats had cannulas implanted during the second half of abstinence). Following recovery, rats were trained to self-administer cocaine (FR train) and then given either short-access (ShA; FR1, maximum of 20 infusions/day, 3 days) or extended-access (ExA; four discrete trials/h, maximum of 96 infusions/day, 10 days) to cocaine self-administration. Then, rats in the ExA group were given access to cocaine in an additional FR1 session (maximum of 20 infusions/day) to verify patency and equate cocaine intake between the ShA and ExA groups prior to withdrawal. Following a 14-day withdrawal period, motivation to obtain cocaine was assessed using a progressive-ratio (PR) schedule (PR SA). Once a baseline level of motivation was established (3 days), the effects of NAc infused eticlopride (0–3 µg/side) were examined. A minimum of three stable PR sessions separated each eticlopride test session. Following study completion, brain tissue was collected for histological confirmation of cannulation placement. Histological cannulae placements in the NAc are shown for **(B)** ShA females, **(C)** ExA females, **(D)** ShA males, and **(E)** ExA males. Schematics adapted from the atlas of Paxinos and Watson.

After recovering from surgery, rats were trained to self-administer cocaine (1.5 mg/kg/infusion; fixed-ratio 1, FR1; maximum of 20 infusions/day) as previously described (Figure 1A; Doyle et al., 2014; Ramôa et al., 2014). Following acquisition (2 consecutive days wherein all 20 infusions were obtained), rats were randomly assigned to either a ShA (*N* = 19 males/20 females) or ExA (*N* = 20 males/16 females) group. Rats in the ShA group were given three additional FR1 training sessions and obtained the maximum number of infusions in each session (20 infusions). Rats in the ExA group were given extended, 24-h/day access to cocaine for 10 consecutive days using a discrete trial procedure (4 trials/hour; up to 96 infusions/day; Doyle et al., 2014; Ramôa et al., 2014) that was designed to mimic the binge-abstinence pattern of drug use observed in humans with CUD (Roberts et al., 2002). Following the last ExA session, rats were given two additional FR1 sessions with a maximum of 20 infusions to minimize differences in cocaine levels between the ShA and ExA groups before withdrawal and to confirm catheter patency. A 14-day withdrawal period began after the last FR1 session for both ShA and ExA rats wherein rats remained in their chambers with the active lever retracted. Following the 14th day of withdrawal, motivation for cocaine (0.5 mg/kg/infusion) was examined under a PR schedule and using methods previously described (Doyle et al., 2014; Ramôa et al., 2014; Towers et al., 2021). These sessions continued daily until a stable baseline was achieved (defined as no increasing or decreasing trend in the number of infusions obtained over three consecutive sessions, typically three to four sessions), then intra-NAc infusions of eticlopride were administered as detailed below.

#### 2.2.3 Effect of Intra-NAc infusion of eticlopride on motivation for cocaine

Once a stable baseline level of motivation for cocaine was established, effects of intra-NAc infusion of the D2 receptor antagonist eticlopride (0.1, 0.3, 1, 3-µg/side) were examined using a within-subject design similar to our previous studies (Doyle et al., 2014; Ramôa et al., 2014; Lynch et al., 2021). Briefly, infusions were administered immediately before the PR test session (0.5 µL/side; 2-min), a minimum of three stable PR sessions separated each test/intra-NAc session, and dose order was counter-balanced between subjects. The eticlopride doses selected were based on our previous intra-NAc infusion study in males showing that these doses either minimally or moderately, but selectively (i.e., no effects on locomotor behavior), impact cocaine self-administration (Schindler and Carmona, 2002; Lynch et al., 2021). Cannula placement was confirmed using methods previously described (Doyle et al., 2014; Ramôa et al., 2014). Placement was within the NAc core (Figures 1B–E) for all but four male and six female rats who were excluded from the study. The final group sizes were 17 for ShA males, 17 for ShA females, 18 for ExA males, and 13 for ExA females. However, due to catheter patency issues, not all rats received all the doses. The final group sizes per treatment (0, 0.1, 0.3, 1, and 3-µg/side) were as follows: 11, 8, 14, 11, and 10 for ShA males, 12, 10, 12, 10, and 12 for ShA females, 9, 9, 10, 8, and 10 for ExA males, and 12, 9, 11, 8, and nine for ExA females, respectively.

### 2.3 Drugs

Cocaine ([-]-cocaine hydrochloride) was obtained from the National Institute on Drug Abuse (NIDA; Research Triangle Park, NC). It was dissolved in sterile saline, filtered, and stored at 4°C. The infusion duration of cocaine was adjusted based on body weight three times per week (2-s/100 g/kg). Eticlopride was purchased from Sigma (St. Louis, MO) and dissolved in sterile water (vehicle).

### 2.4 Data analysis

Sex differences in cocaine intake over the 10-day ExA period were examined using a mixed effects model with sex as the between-subject factor. A mixed effect model was also used to assess for the development of an addiction-like phenotype in the ExA group, which was defined by an enhanced motivation for cocaine relative to the ShA group. This analysis focused on the group differences (i.e., ShA females, ShA males, ExA females, ExA males) in the number of infusions obtained during the three PR baseline sessions that preceded the first treatment. Given baseline differences in the number of cocaine infusions obtained between the ExA and ShA groups and males and females, group differences in the effects of NAc-eticlopride infusion were analyzed as percent change from baseline using group (i.e., ShA females, ShA males, ExA females, ExA males) and treatment (saline *versus* D2 receptor antagonism) as fixed factors and dose (0–3.0 µg/side) as a covariant. Post hoc comparisons to baseline (0) were made using two-tailed Bonferroni-corrected one sample t-tests. Statistical analyses were performed using SPSS (V26). Alpha was set at 0.05. Data are presented as the mean ± SEM.

## 3 Results

### 3.1 Cocaine self-administration and subsequent motivation for cocaine

**FIGURE 2:**
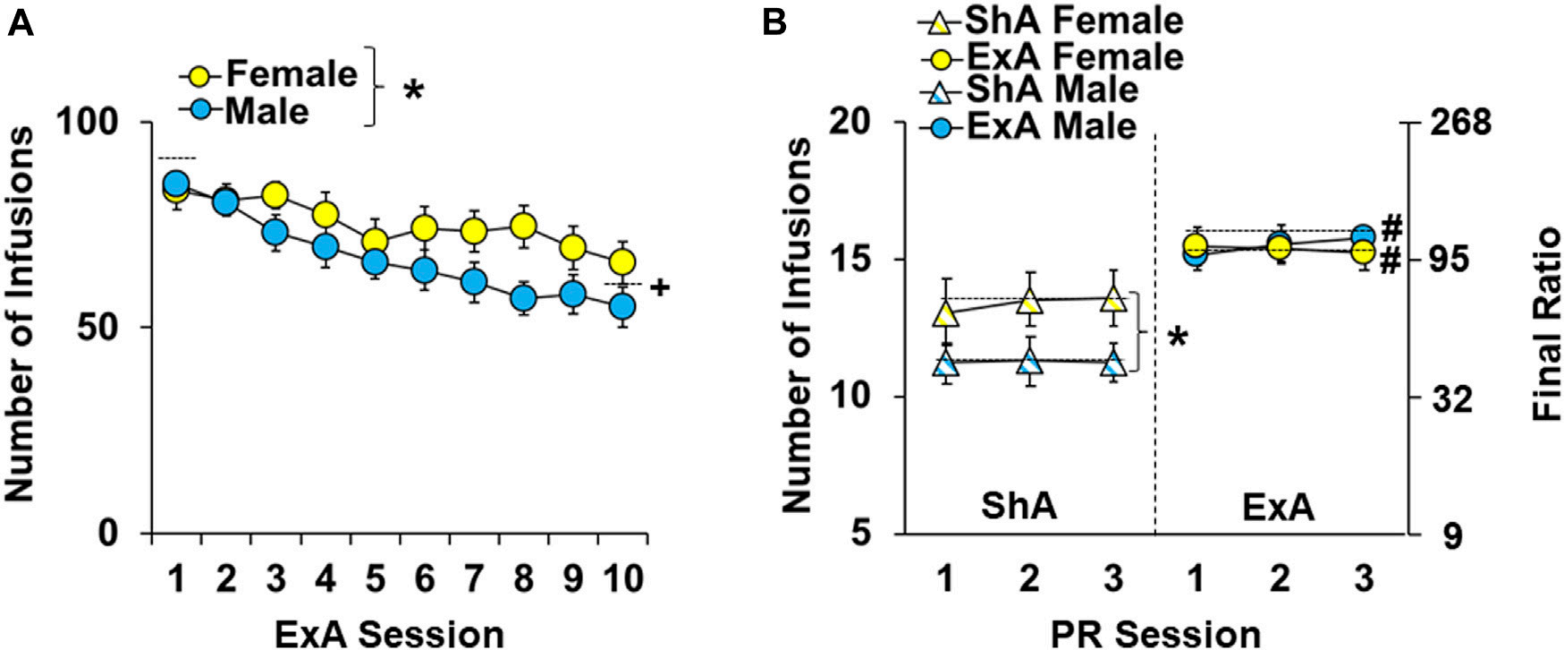
Sex differences in the impact of extended-access cocaine self-administration and withdrawal on motivation for cocaine. Mean (±) number of infusions obtained during the 10 extended-access (ExA) cocaine self-administration sessions **(A)** and the first three stable progressive ratio sessions **(B)** for ExA males (*n* = 18) and females (*n* = 13) and short-access (ShA) males (*n* = 17) and females (*n* = 17). ∗ significant difference between males and females. # significant difference between the ShA and ExA group. Dashed line indicates the data included in each comparison.

Females self-administered significantly more cocaine infusions under ExA conditions than males (Figure 2A; overall effect of sex: F_1,300_ = 16.2, *p* < 0.001); however, females and males had a similar pattern of cocaine self-administration over the 10-day ExA period (no session by sex interaction) with both sexes obtaining more infusions during early sessions compared to later sessions (overall effect of session: F_9,300_ = 5.7, *p* < 0.001; session one *versus* 10, *p* < 0.001). Additionally, the average number of infusions obtained by ExA females and males over the 10-day ExA period (75 ± 1 infusions/session and 67 ± 2 infusions/session, respectively) was significantly higher than the number of infusions obtained by ShA males and females which obtained 20 infusions per session (data not shown). Thus, while ExA females had higher cocaine intake than ExA males, both sexes had a similar pattern of cocaine intake over the ExA period and had higher intake than ShA controls.

As expected, following the 14-day withdrawal period, ExA rats obtained more cocaine infusions during the three PR baseline sessions that preceded the first eticlopride infusion than ShA controls (Figure 2B overall effect of group: F_3,183_ = 19.5, *p* < 0.001). Post-hoc comparison within each sex confirmed that ExA rats obtained more cocaine infusions than ShA rats for both males (*p* < 0.05) and females (*p* < 0.05). ShA females also obtained more cocaine infusions than ShA males (*p* < 0.05); however, there was no difference in the number of infusions obtained between ExA females and males (*p* < 0.05). There were also no overall or interactive effects of session, indicating that baseline levels of motivation were stable within each group. Thus, while females had higher motivation for cocaine than males following ShA cocaine self-administration, both males and females were more motivated to obtain cocaine following withdrawal from ExA self-administration (relative to ShA controls), which confirms the development of an addiction-like phenotype in both sexes.

### 3.2 Effects of NAc-D_2_ antagonism on motivation for cocaine

**FIGURE 3:**
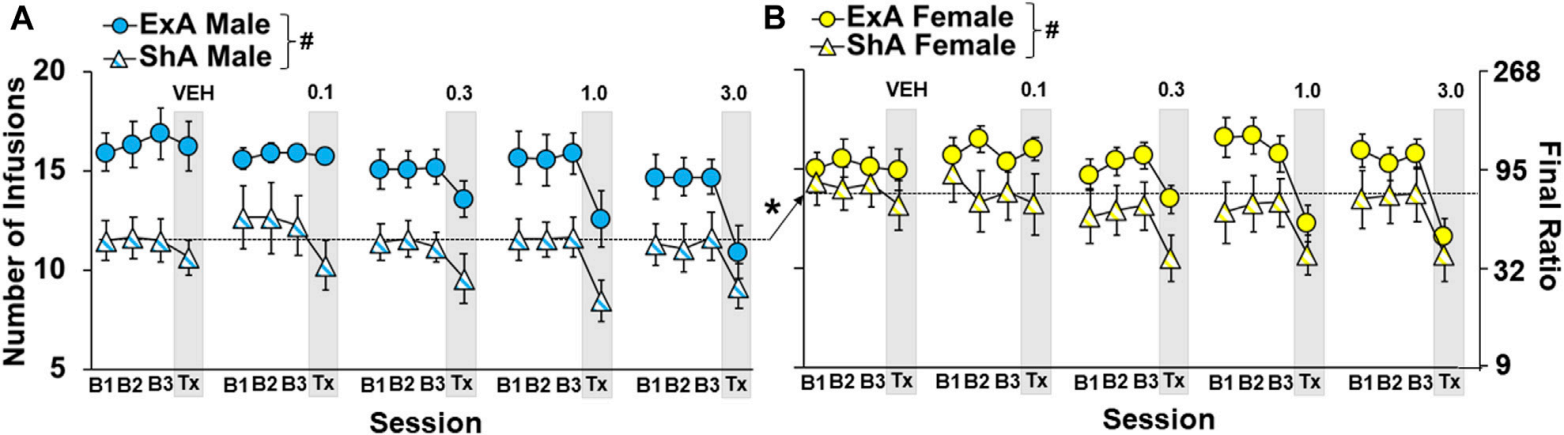
Group differences in baseline levels of motivation for cocaine were maintained throughout the study. Mean (±) number of infusion obtained during the three stable progressive ratio (PR) sessions prior to treatment (B1, B2, B3) and the day of treatment (Tx; grey box) as a function of eticlopride dose (0, 0.1, 0.3, 1.0, and 3.0 -µg/side) in short-access (ShA) (*n* = 11, 8, 14, 11, 10) and extended-access (ExA *n* = 9, 9, 10, 8, and 10) males **(A)** and ShA (*n* = 12, 10, 12, 10, 12) and ExA (*n* = 12, 9, 11, 8, 9) females **(B)**. ∗ significant difference between males and females. # significant difference between the ShA and ExA group. Dashed line indicates the data included in each comparison.

Motivation for cocaine was stable for the three baseline sessions that preceded each treatment session across the study and this level of motivation was re-established following each treatment session for each group (no overall or interactive effects of dose: *p* > 0.05 s). However, as with the analysis on the number of infusions obtained prior to the first eticlopride infusion, group differences were observed for the number of infusions obtained in the three baseline sessions that preceded each treatment session (overall effect group: F_3,558_ = 41.7, *p* < 0.001; Figures 3A,B). This difference was driven by higher cocaine infusions in the ExA *versus* ShA group (*p* < 0.001 s) and in ShA females compared to ShA males (*p* < 0.001).

**FIGURE 4:**
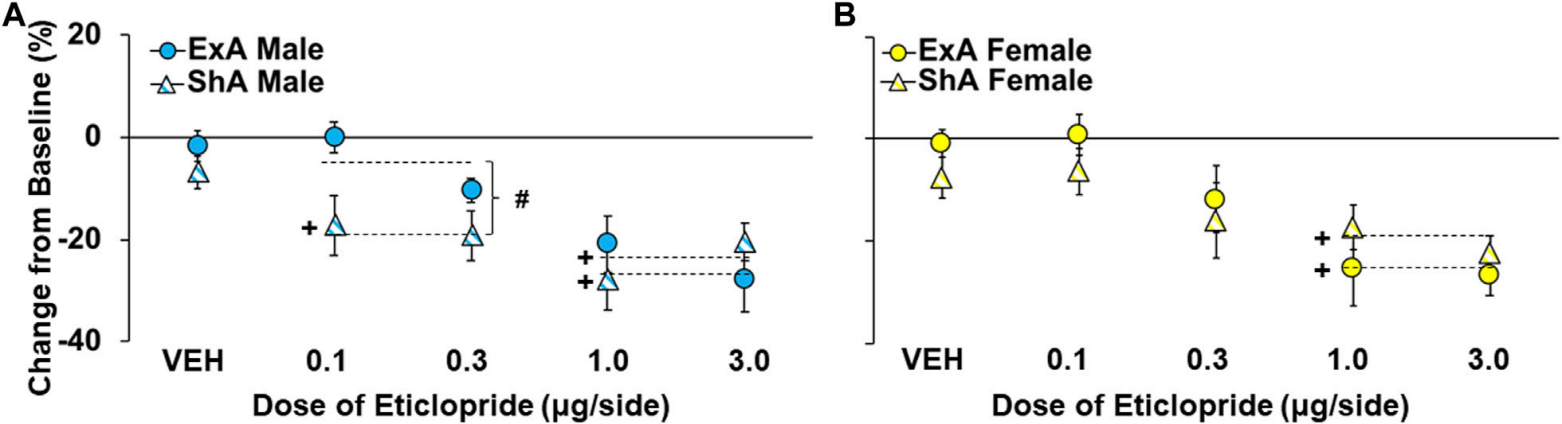
Sex differences in effects of NAc-D2 receptor antagonism on motivation for cocaine prior to and following the development of an addiction-like phenotype. Mean (±) percent change from the baseline number of infusions obtained by short-access (ShA) and extended-access (ExA) groups following treatment with eticlopride (0, 0.1, 0.3, 1.0, and 3.0 -µg/side) in males **(A)**; ShA, *n* = 11, 8, 14, 11, 10; ExA *n* = 9, 9, 10, 8, and 10) and females **(B)**; ShA, *n* = 12, 10, 12, 10, 12; ExA *n* = 12, 9, 11, 8, 9). # significant difference between the ShA and ExA group. + significant decrease from baseline (0). Dashed line indicates the data included in each comparison.

Given these baseline group differences in PR responding, effects of D_2_ receptor antagonism were determined as percent change from the average number of cocaine infusions obtained during the three baseline PR sessions that preceded each treatment (Figures 4A,B). This analysis indicated that greater effects were obtained at higher *versus* lower eticlopride doses (overall effect of dose: F_1,194_ = 32.6, *p* < 0.001) and in the ShA *versus* ExA group (overall effect of group: F_3,194_ = 3.7, *p* < 0.05; posthocs *versus* ShA males), particularly in males at low doses of eticlopride (interaction of group and dose: F_3,194_ = 2.7, *p* < 0.05). Further analysis within males confirmed significant effects of group (F_1,95_ = 8.0, *p* < 0.01) and group by dose (F_1,95_ = 6.0, *p* < 0.05), which was driven by greater decreases in the ShA *versus* ExA group, particularly at lower doses (0.1 and 0.3 µg/side; *p* < 0.01). There was also a significant effect of dose (F_1,95_ = 13.5, *p* < 0.001) indicating that, as with the larger group, higher doses induced larger decreases. Additionally, *post hoc* comparisons to baseline (0) within each group of males confirmed a significant decrease in motivation for cocaine use in the ShA group at both the low doses (0.1 and 0.3 µg/side: *p* < 0.001) and high doses (1.0 and 3.0 µg/side: *p* < 0.001), but not vehicle (*p* > 0.05); whereas, in the ExA group, only the effect at the high doses reached statistical significance (*p* > 0.001); vehicle was not significant (*p* > 0.05), and the low doses only tended to decrease motivation for cocaine (*p* = 0.06).

In contrast to effects in males, the analysis within females revealed only a trend for greater effects in the ShA *versus* ExA group (overall effect of group: F_1,99_ = 2.8, *p* = 0.10) and no significant interaction of group and dose (*p* > 0.05). Additionally, further analysis within the two low doses (0.1 and 0.3 µg/side) and two high doses (1.0 and 3.0 µg/side) of eticlopride revealed a similar decrease in motivation for cocaine use in ShA and ExA females (no group effect: *p*’s > 0.05). There was also a significant effect of dose (F_1,95_ = 13.5, *p* < 0.001), indicating that, as with the larger group and findings in males, higher doses induced larger decreases in motivation for cocaine use. Post-hoc comparisons to baseline (0) within each group of females confirmed the only significant decrease in motivation for cocaine use was at the two high doses in ShA and ExA females (*p*’s < 0.001); whereas, vehicle was not significant in ShA and ExA females and the low doses tended to decrease motivation for cocaine use in ShA females (*p* = 0.054), similar to ExA males. Thus, while NAc-infused eticlopride dose-dependently decreased motivation for cocaine following withdrawal from both ShA and ExA cocaine self-administration and in both males and females, the effect was most robust in ShA males, particularly at low doses.

## 4 Discussion

We previously showed that an addiction-like phenotype, defined as the development of enhanced motivation for cocaine, develops over withdrawal following ExA cocaine self-administration and, in males, corresponds with a diminished role of NAc-D2 receptor signaling (Lynch et al., 2021). In the present study, we expanded the investigation of D2 receptor mechanisms motivating cocaine use to include females. Based on findings in human smokers, we predicted that the role of NAc-D2 receptor signaling would vary between males and females and in females, would not become diminished with the development of an addiction-like phenotype. Our findings support this hypothesis as we found that eticlopride was less effective at decreasing motivation for cocaine in ExA males *versus* ShA males, particularly at low doses; whereas, in females, there were no differences in the effectiveness of the eticlopride between ExA and ShA rats. These findings indicate that males, but not females, become less sensitive to NAc-D2 receptor antagonism with the development of an addiction-like phenotype.

Decreased striatal D2 receptor binding in addiction is one of the most replicated findings in human imaging research (see Trifilieff et al., 2017 for review); however, this molecular shift may not translate to females (Brown et al., 2012; Zakiniaeiz et al., 2019). Our findings in the present study build on this possibility by showing that while low doses of eticlopride were less effective at decreasing motivation for cocaine in ExA *versus* ShA males, there was no difference in its efficacy between ExA and ShA females (Lynch et al., 2021). Based on the assumption that low doses are more selective and induce fewer off target effects than high doses, our findings indicate that males, but not females, develop a decrease in sensitively to D2 receptor antagonism with the development of an addiction-like phenotype. One alternative possibility, however, is that this shift does occur in females, but perhaps sooner/more readily, such that it was already present in females tested following ShA self-administration. Females are known to transition from initial drug use to meeting the criteria for CUD/seeking treatment faster than males (for review see Towers et al., 2023). Additionally, in our present study, ShA females were more motivated to obtain cocaine than ShA males. Thus, it is possible that at least some of the females in the ShA group had already developed an addition-like phenotype and a shift toward a diminished role of D2 receptors and this explains why we did not see a difference between ShA and ExA females. It is notable that no differences were observed for the effects of eticlopride between ExA males and females. Further research using a within subject design to determine the development of an enhanced motivation for cocaine (relative to baseline prior to ShA *versus* ExA self-administration) is necessary to resolve this question.

It is also important to note that findings in this study along with ours (Doyle et al., 2014; Ramoa et al., 2014) and others previous studies (e.g., Castner et al., 2000; Henry et al., 2009) provide support for similarities in the mechanisms underlying addiction in males and females. More specifically, here we showed NAc-D2 receptor blockade with eticlopride dose-dependably decreased motivation for cocaine in both ShA and ExA males and females. This indicates that D2 receptors remain critical for motivating cocaine use prior to and following the development of an addiction-like phenotype in both sexes. This is in contrast to our previous findings for NAc dopamine 1 (D1) receptor signaling, which showed NAc-D1 receptor blockade with SCH-23390 dose-dependably decreased motivation for cocaine in ShA, but not ExA, males and females. These findings indicate that D1 receptors are critical for motivating cocaine use prior to, but not following, the development of an addiction-like phenotype in both sexes (Doyle et al., 2014; Ramoa et al., 2014). Preclinical studies in non-human primates have also shown stimulant-induced dopamine release from presynaptic terminals is decreased in males and females with repeated drug use (Castner et al., 2000; Henry et al., 2009). Thus, blunted dopamine transmission in the striatum likely renders males and females vulnerable to continued drug use, and there are likely similarities (D1R signaling) and differences (D2 receptor signaling) in the mechanisms underlying these striatal dopamine deficits associated with addiction in males and females.

There is an urgent need to disentangle the differences and similarities in the mechanisms underlying CUD in males and females. While males having higher rates of cocaine use than females, the number of females using cocaine and other addictive drugs in their lifetime is on the rise, and females are more vulnerable to many aspects of CUD compared to males. For example, females develop CUD and/or seeking treatment for the disorder after few years of cocaine use, report longer periods of cocaine use after relapse, have greater stress-induced cravings, and show an enhanced vulnerability to cocaine-related medical consequences compared to males (Griffin et al., 1989; White et al., 1996; McCance-Katz et al., 1999; Haas and Peters, 2000; Gallop et al., 2007; Waldrop et al., 2010; Moran-Santa Maria et al., 2018). Preclinical studies have similarly shown that females have an enhanced sensitivity to reinforcing effects of cocaine, and display a greater vulnerability during the transition from initial cocaine use to the development of an addiction-like phenotype, including self-administering more cocaine under ExA conditions and developing an enhanced motivation for cocaine/preference for cocaine over other reinforcers after less drug exposure and/or shorter periods of withdrawal than males (see Towers et al., 2023 for review). Although the present study was not designed to assess behavioral sex differences, similar to the findings mentioned above, we observed higher levels of cocaine intake under ExA conditions and motivation for cocaine use following ShA cocaine self-administration in females than males. Thus, females and males also differ in several key features of addiction, and understanding sex differences in the mechanisms underlying these features will hopefully lead to the development of sex-specific treatments and the first FDA approved treatment for CUD.

In summary, despite the severity of addiction in females and an accumulating body of evidence indicating that females and males differ in several features of addiction, clinical and preclinical studies have historically used male subjects, resulting in a male-centric neurobiological basis of addiction. The current study builds on the literature showing that findings in males do not necessarily translate to females. Future studies are needed to and to determine whether striatal D2 receptor signaling becomes diminished with the development of other addiction-like features in females, such as compulsive use despite negative consequences and preferential use of cocaine over other natural rewards, as well as with the development of other substance use disorders such as opioid use disorder. Future studies are also needed to determine the impact of ovarian hormones on D2 signaling in females especially considering that ovarian hormones, and particularly estradiol, is known to impact cocaine’s reinforcing effects and the development of addiction-like features in females (see Towers et al., 2023 for review).

## Data Availability

The raw data supporting the conclusion of this article will be made available by the authors, without undue reservation.
